# Case report: Solitary fibrous tumor of the kidney with a NAB2-STAT6 fusion gene

**DOI:** 10.3389/fonc.2022.1045238

**Published:** 2022-11-16

**Authors:** Wen-Tong Ji, Yu Hu, Yao Wang

**Affiliations:** ^1^ Urology 2nd Department, China-Japan Union Hospital of Jilin University, Changchun, Jilin, China; ^2^ Pathology Department, China-Japan Union Hospital of Jilin University, Changchun, Jilin, China

**Keywords:** case report, solitary fibrous tumor, immunohistochemical assay, genetic testing, NAB2-STAT6

## Abstract

**Background:**

Solitary fibrous tumor (SFT) is a rare spindle cell neoplasm that mostly originates from the pleura, and accounts for only 2% of all soft tissue tumors. Moreover, the cases of SFT of the kidney are rarely reported. Here, we report a typical case of kidney SFT, which was consistent with other reported cases. This case further expands on existing diagnostic methods of SFT and explains the importance of STAT6 mutations in SFT.

**Case Summary:**

We report a typical case of SFT of the kidney. A 34-year-old woman presented to the urinary surgery department after physical examinations were suggestive of a urologic neoplasm. Further relevant imaging investigations suggested a renal tumor with benign behaviors. The patient was diagnosed with a kidney tumor suspected to be SFT and underwent laparoscopic radical left nephrectomy. Postoperative pathological immunohistochemical tests showed positivity for Signal Transducer and Activator of Transcription 6(STAT6), CD-34, CD-99, and Bcl-2, thus confirming the diagnosis of SFT. Combined with the results of genetic testing of the patient, the tumor was indicated to carry NGFI-A-Binding protein 2(NAB2): exon 6—STAT6: exon 16 mutation sites, which confirmed our diagnosis. The patient recovered quickly without any clinical evidence of incomplete resection. She has been followed-up for more than a year and will continue to be reviewed every three months to observe the final outcomes.

**Conclusion:**

Solitary fibrous tumor is difficult to differentiate from other renal tumors. CT imaging, STAT6 immunostaining and gene profiling are valid investigations to establish the diagnosis.

## Highlights

SFT of the kidney are rarely reported worldwide, and systematic principles of diagnosis and treatment are still to be established. Hence, we present a typical case of SFT of the kidney to confirm the generality of existing cases. We analyzed the outcomes of imaging investigations and pathological examinations, and indicated potential areas for future research. We also speculate on the molecular mechanism of SFT arising from the transcription process.

## Introduction

Solitary fibrous tumor(SFT) is a rare mesenchymal neoplasm usually found in the pleura, accounting for only 2% of all soft tissue tumors (1) and it is occasionally accompanied by paraneoplastic syndromes (2). SFTs of the kidney occur more rarely compared with those originating from the pleura and demonstrate a different clinical behavior. Generally, a SFT is difficult to differentiate from other malignant renal tumors; hence, most patients with SFT of the kidney are erroneously diagnosed with renal carcinoma. Here, we provide a representative case, highlighting most of the clinical features of this disease. The patient underwent radical nephrectomy and we conducted a series of pathological examinations and gene testing with the agreement of the patient. The results of these examinations confirmed the results of previous cases. Furthermore, herein, we also discuss Signal Transducer and Activator of Transcription 6(STAT6) and NGFI-A-Binding protein 2(NAB2)-STAT6 fusion gene further. The specific contents are written in the following case presentation.

## Case description

### Chief complaints

A 34-year-old woman was admitted to the Urology 2nd department of China-Japan Union Hospital for further examination due to a space-occupying lesion in the left kidney, revealed by urinary ultrasonography performed on physical examination.

### History of present illness

The patient had no complaints of illness. A left kidney mass was found by color ultrasonography performed during routine physical examination.

### History of past illness

Previous cardiac radiofrequency ablation was performed in the Department of Cardiology due to frequent ventricular premature beats with partial second and third rhythms.

### Personal and family history

No significant personal or family history was reported.

### Physical examination

No other abnormality was revealed on physical examination.

### Laboratory investigations

No significant abnormalities were revealed on laboratory examination.

### Imaging investigations

**Figure 1:**
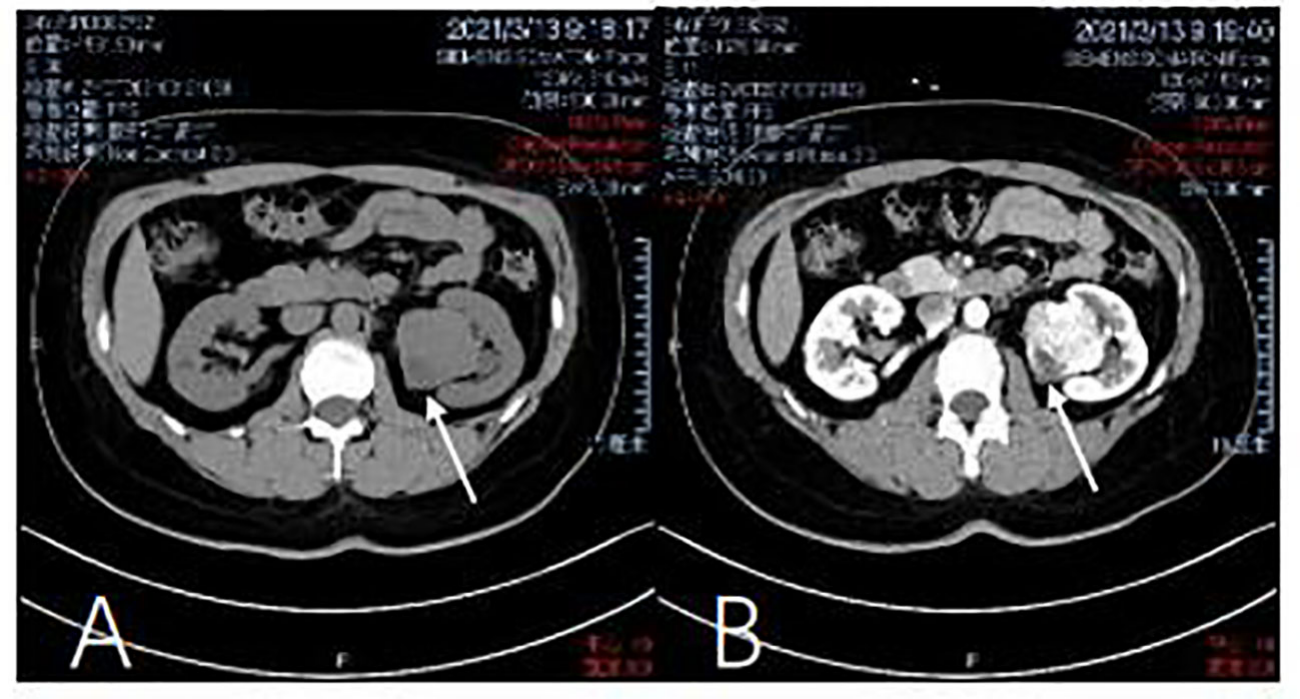
Computed tomography of the kidney. **(A)** Abdominal pelvis CT scan shows a mass in the left kidney. There is a no dular mass of about 4 x 3.3 x 3 cm in the middle and lower pole of the kidney. (March 13, 2021). **(B)** Contrast-enhanced CT shows heterogeneous enhancement in the cortical phase (March) 13, 2021).

As shown in **Figure 1**, a computed tomography (CT) scan of the abdomen and pelvis in March, 2021, showed a mass-like high density shadow arising from the anterior cortex, beside the left renal pelvis, about 4.1 × 4.5 cm in size, well-demarcated, with benign features. For example, the tumor tissue showed no invasion into peripheral structures, especially the renal vessels and peri-renal adipose tissue. The left renal pelvis was slightly deformed under the extrusion of the tumor, but the vena cava was normal. No intra-tumoral calcification was observed. Near the capsule of the tumor, a round area showed relatively low density, which suggested cystic degeneration, hydrops, or hemorrhage. Contrast-enhanced CT showed heterogeneous enhancement in the cortical phase. The tumor was noted strong enhancement in both cortical phase and delayed phase, which illustrated that the tumor has abundant blood supply. In comparison, a renal malignant tumor would be rapidly filled up by blood and the image of the cortical phase would be brighter. A heterogeneous pattern of increased attenuation would be noticed in this phase, and the enhancement should be slightly decreased from surrounding parenchyma. However, the nodular mass had peripheral enhancement and fluid collection in the center. Hence, based on the CT image, the tumor was more likely to be an SFT rather than a malignant tumor. But the tumor showed heterogeneous enhancement in the cortical phase, which was difficult to be distinguished from the malignancy. So in preoperative stage, we still could not rule out the possibility of malignancy totally.

### Diagnostic assessment

The process of diagnosis is difficult, because the results of laboratory investigations were negative and we could not distinguish SFT with other tumors in the very beginning.

### Final diagnosis

Left kidney SFT.

### Treatment

The patient underwent laparoscopic radical nephrectomy of the left kidney under general anesthesia, and was grossly sent for pathology after surgery.

### Findings in pathological examination

**Figure 2:**
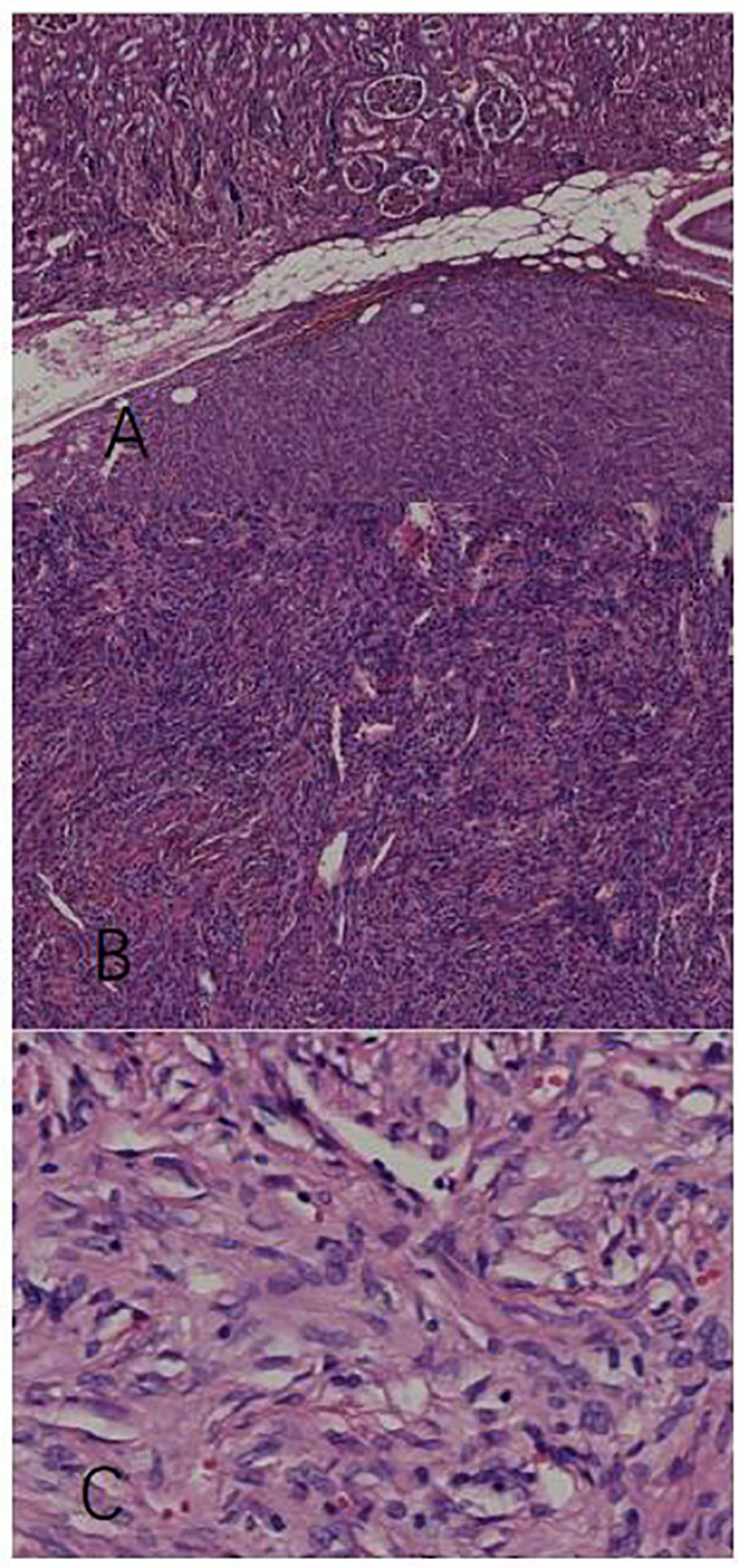
Pathological examination with hematoxylin-eosin stain. **(A)** The tumor tissue has a clear boundary with the intact parenchymal issue (x40). **(B)** The tumor tissue contains compactly arranged cells a nd blood vessels of different shapes (x100). **(C)** Spindle tumor cells row with benign patterns (x400).

We used hematoxylin-eosin staining to stain the tissue sample. A mass of the normal tissue from the parenchyma is attached to the tumor tissue for comparison. **Figure 2A** shows that the tumor tissue is clearly demarcated from the normal renal tissue. On magnification of the tumor tissue in **Figure 2B**, we find that the tissue loses the normal microscopic structure of a renal tissue. Cells were arranged like stripes, fish-bones, and even vortexes. The whole zone could be divided into several areas of cell-concentration and cell-sparsity. In cell-concentration areas, cells were spindle-shaped or oval. Most of the cell space was taken up by nucleus. And in cell-sparsity areas, cells were like slender spindles. In both area, cells showed no obvious atypia and anomalous nuclei were rare. Meanwhile abundant blood vessels were observed among tumor cells. The vessels varied in caliber and size; some expanded to form sinuses, and some formed crevices and antlers. Under x400 magnification (**Figure 2C**), the tumor cells were spindle-like with ovoid nuclei, small nucleoli, lightly-stained cytoplasm, and unclear boundaries. The intracellular space was filled with dense collagenous bands. The patterns of the tumor tissue were in accordance with a typical SFT (3) The morphology of the nucleus was normal and the cells showed benign behavior.

### Findings in immunohistochemical staining

**Figure 3:**
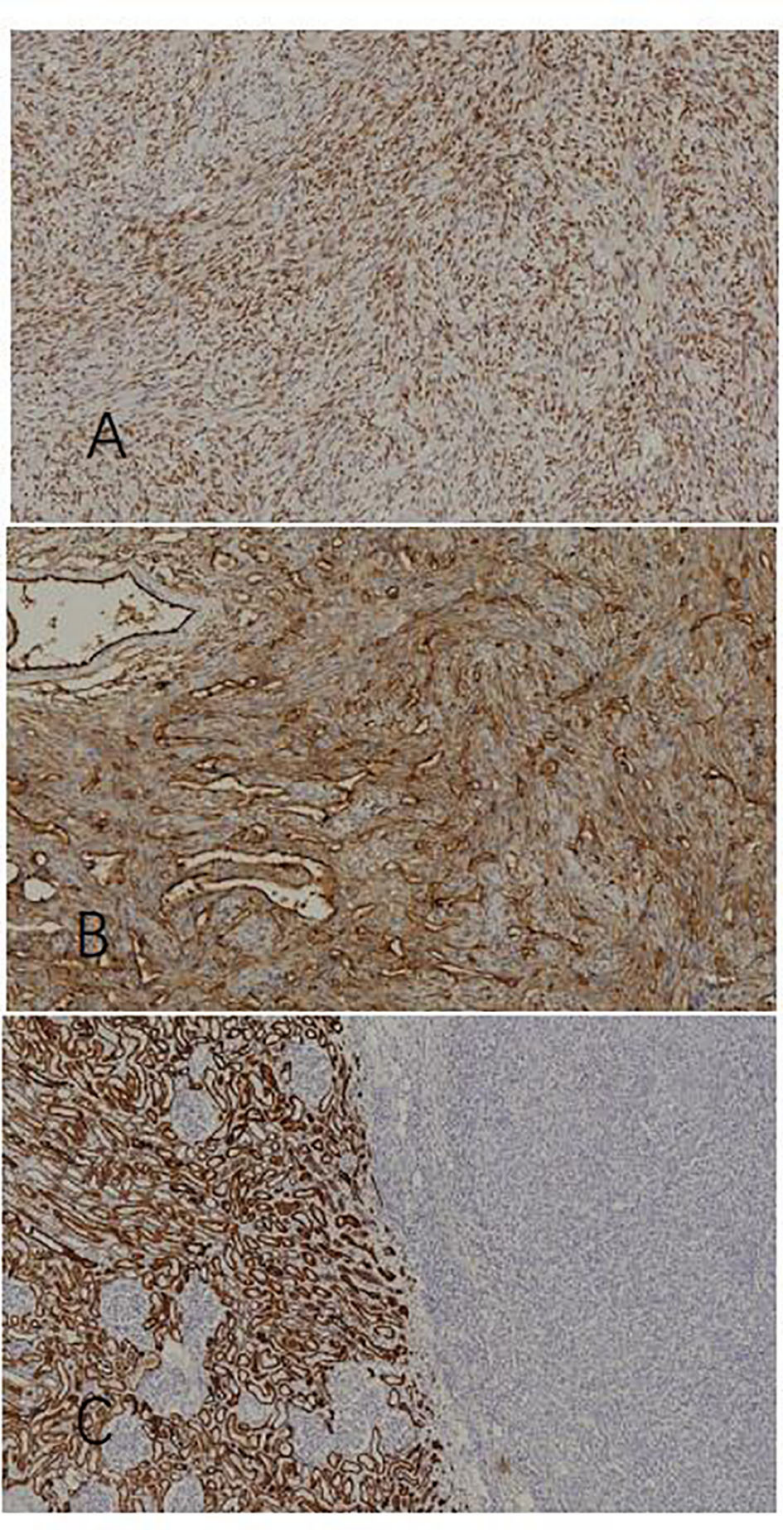
Immunohistochemical staining. **(A)** STAT6(+) (x100), **(B)** CD34(+)(x100), and **(C)** normal renal tissue in the left part: CKpan(+); tumor tissue in the right part: CKpan(-)(x100).

The diagnosis of SFT of the kidney can be confirmed by pathological immunohistochemistry: STAT6 (+), CD34 (+), Bcl-2 (+), vimentin (+), Ki67 (3% +), S-100 (-), SMA (-), PAX-8 (-), EMA (-), CKpan (-), and CD117 (-). As shown in **Figure 3**, STAT6(+) and CD34(+) have diagnostic significance. Additionally, the tumor cells and endothelial cells of the blood vessels are both positive for CD34, which illustrate that the two groups of cells have definite homology. Lastly, CKpan(-) demonstrates the heterology between the tumor and renal parenchymal tissue.

### Findings in gene testing

**Figure 4:**
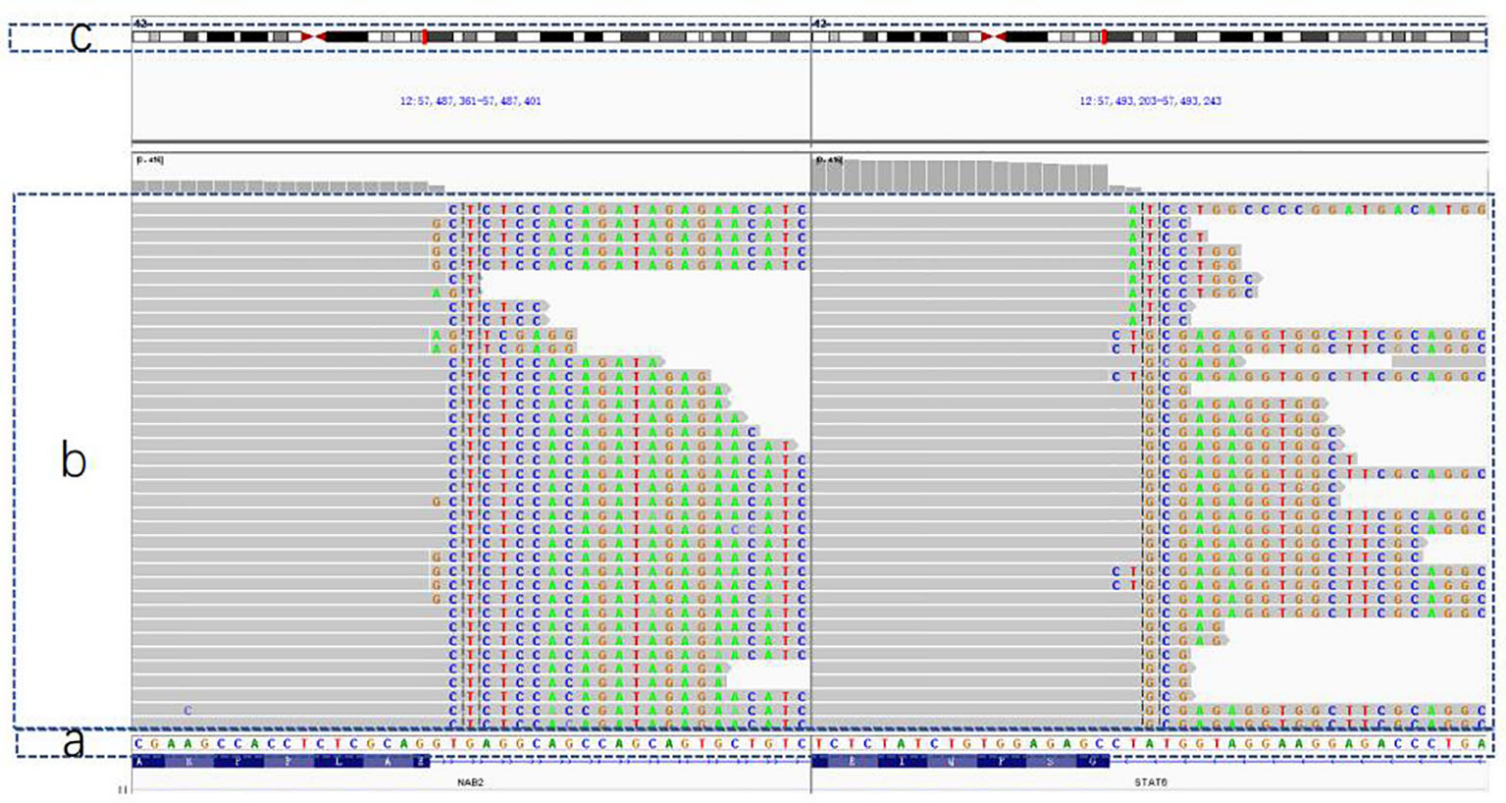
The outcome of genetic testing with GRCh37/hg19 genome for reference. **(A)** The normal gene sequence for contrast. **(B)** The patient's gene: gene orders in the gray part are in accordance with **(A)** sequence and faulty orders are highlighted in different colors **(C)** A simple diagram of chromosome No. 12, which was thought to be broken in two sites and fused together so that the NAB2-STAT6 mutation came into being.

After obtaining informed consent from the patient, we took the patient’s pathological specimens for genetic testing. Next Generation Sequencing(NGS) method was used under Illumuna HiSeq 4000 High-Throughput Sequencing platform. Genome sequencing was accomplished and the outcome of genome sequencing was referred to GRCh37 and hg19 genome, which were two versions of reference genome. In this process, we recorded all the detectable mutations in the sequencing. Then, by comparing the mutations with the Single Nucleotide Polymorphism Database(dbSNP), we eliminated irrelevant mutations and merely analyzed SFT-specific mutations. As shown in **Figure 4**, mRNA was reverse transcribed into DNA; we found that chromosome 12 harbored NAB2 (NGFI-A binding protein 2)-STAT6 fusions, which was located in the NAB2: exon 6—STAT6: exon 16 (accounted for 94.4%) by contrasting with a template strand of STAT6 mRNA. Oddly, however, we used DNA to construct a genomic sequencing in the beginning but we did not find any mutation related to the occurrence of SFT. Then we used RNA fragments and constructed one by reverse transcription. So consequently, NAB2-STAT6 fusion gene was found.

### Outcome and follow-up

Through phone call following-up we knew that the patient was satisfied with the result of the treatment. She recovered well without complications after surgery. The patient was asked to follow-up with abdominal CT scans, with contrast, every three months for surveillance, but to date, there have been no clinical signs or symptoms of recurrence, nor any clinical evidence of incomplete resection.

## Discussion

SFT has many similar phenotypes with hemangiopericytomas. Specifically, CD-34, CD-99, Bcl-2, and STNT6 are considered as markers of both tumors (4). STF usually occurs in the pleura. Most SFTs progress slowly with relatively good prognosis, but malignant cases occur in 10% of all cases (4).The probability of SFTs occurring in the kidney is low, but owing to its rarity, the process of diagnosis and treatment is challenging (5). Some recorded cases show that the pathognomonic gene fusion between NAB2 and STAT6 is highly sensitive and specific for SFTs (6).

Combined with the diagnosis and treatment of this case, it can be found that SFT of the kidney is difficult to detect early and mainly depends on CT to differentiate it from malignant renal tumors. In our case, the outcome of pathological examination and immunohistochemical staining showed typical characteristics of STF. But both are not optimal to use in preoperative stage. The patient was followed-up and did not show any clinical signs, symptoms, or clinical evidence of incomplete resection. The disease is therefore considered to have a good prognosis. According to the relevant literature, it is found that the majority of SFT diagnoses are based on postoperative pathological immunohistochemistry or preoperative renal biopsy. At present, strong CD34 and STAT6 reactivity is considered to be characteristic and is indispensable in the diagnosis of SFT. Although traditionally, CD34 (+) is the most consistent reported finding in SFT, present in up to 95% of cases, it also appears in many other tumors (7), making it non-specific in SFT. Therefore STAT6 is more reliable in the diagnosis of SFT. Combined with the results of genetic testing in this patient, it is further demonstrated that there is a close relationship between STAT6 (+) and SFT.

STAT6 belongs to a family of transduction factors and STAT6 is related to the function of the immune response and affects the progression of allergic inflammation (8). Overexpression of STAT6 plays an important role in the generation and progression of lymphoma, especially Hodgkin’s lymphoma (9). Therefore doctors should distinguish SFT of the kidney from renal lymphoma when they encounter a patient with STAT6(+) tumors. In the above-mentioned condition, we believe genetic testing is the best way to differentiate SFT of the kidney from other STAT6-related lymphomas. Furthermore, Koelsche et al. illustrate that the gene fusion of NAB2 with STAT6 is the responsible event at the molecular level; therefore, NAB2-STAT6 fusion discovery in genetic testing helps to discriminate SFTs from other histological mimics (10). Karpathiou et al. also point out that the overexpression of STAT6 exerts different effects on SFT and lymphoma (11). The specific function of STAT6 in the occurrence and development of SFT still needs further research.

NAB2-STAT6 fusion gene consists of an integrated domain of STAT6 and a truncated repressor domain of NGFI-A-Binding protein 2 (NAB2) (12). NAB2 is a protein that can repress the transcription of early growth response protein-1 (EGR1), And EDR1 is evaluated as a tumor-suppressor (13). In NAB2-STAT6 fusion gene, NAB2’s function of indirectly inhibiting tumor growth is impaired but the function of STAT6 is still intact. On this basis, it can be assumed that STAT6 domain still produces STAT6 protein in our case.

Meanwhile it is important to point out that STAT6 variants contains two fusion groups, STAT6-Full and STAT6-TAD. A relevant study demonstrated that recurrence-free interval and overall survival rates are different between the STAT6-Full group and STAT6-TAD group. Patients from the STAT6-TAD group have better prognosis and tyrosine kinase inhibitors show better efficacy in the treatment of this group (14), which suggests that radical nephrectomy may not be the only method for treating SFT. More clinical experiments need to be carried out to find the optimal treatment for each group. According to statistics, SFTs in meninges with NAB2: exon 6—STAT6 have higher recurrence rates (50%) compared with SFTs with NAB2: exon 4—STAT6 (27%) (4), but the influence of NAB2: exon 6—STAT6 in kidney SFTs on recurrence is still unknown. Due to the lack of a large number of valid data, more related studies are needed to further confirm the fusion mutation site of NAB2: exon 6—STAT6: exon 16 that led to STAT6 in this case. Thus, when analyzing the negative result of DNA sequencing in this case, we speculate that genetic information is altered in the process of transcription. As a result, the fusion mutation can only be found through reverse transcription using faulty mRNA.

In preoperative stage, we lacked diagnostic methods and we mainly depended on imaging examination. We found that the tumor has abundant blood supply and the size of it is relatively large. Given that the proximity of the tumor to renal hilus, nephron-sparing surgery would be riskier and more difficult. Although the tumor showed no invasion into peripheral tissue, we still could not rule out the possibility of malignancy. After taking the interests of the patient into full consideration, we chose laparoscopic radical nephrectomy of the left kidney. Now when reviewing the whole process of our treatment, we think nephron-sparing surgery is also suitable.

In our case, the patient recovered without complications and we found no sign of recurrence in follow-up treatment. However, Ichiyanagi et al. proposed that SFT still has the potential of malignant transformation although its behavior is benign (15). Moreover, subsequent recurrence and even distant metastasis are also possible after resection (16). We will, therefore, keep track of this case.

## Conclusion

First reported in 1931, there are 111 reported cases of SFT of the Kidney worldwide up to now. Gene sequencing was carried out on a small fraction of them. Among these who have been sequenced, there was one reported case of SFT of the kidney with NAB2-STAT6 fusion gene before. Our case confirmed the generality of SFT of kidney existed in other reported cases, further expanded the existing database of this tumor. We used imaging, pathological examination, immunohistochemical staining and gene testing to find particularity and commonality of our case compared with other existing cases. It also emphasized the importance of examination and gene sequencing. Hopefully, this will push forward the understanding of this tumor and advance the research into SFT of the kidney.

## Data availability statement

The original contributions presented in the study are included in the article/supplementary material. Further inquiries can be directed to the corresponding author.

## Ethics statement

The studies involving human participants were reviewed and approved by China-Japan Union Hospital of Jilin University. The patients/participants provided their written informed consent to participate in this study.

## Author contributions

W-TJ and YH performed the investigations; W-TJ wrote the manuscript; YW acquired the funding; and YW contributed to review and editing of the manuscript. All authors contributed to the article and approved the submitted version.

## Funding

This study was supported by Basic Research Project of Jilin Provincial Department of Science and Technology No. 20190201060JC.

## Conflict of interest

The authors declare that the research was conducted in the absence of any commercial or financial relationships that could be construed as a potential conflict of interest.

## Publisher’s note

All claims expressed in this article are solely those of the authors and do not necessarily represent those of their affiliated organizations, or those of the publisher, the editors and the reviewers. Any product that may be evaluated in this article, or claim that may be made by its manufacturer, is not guaranteed or endorsed by the publisher.
